# Early Toxicities of CAR-T Cell Therapy: Mechanisms, Prediction, and Management

**DOI:** 10.3390/ijms27219587

**Published:** 2026-10-27

**Authors:** Swathi Srikantan, Alexander Weiss, Zainab Muslehuddin, Shatha Farhan

**Affiliations:** 1 School of Medicine, Wayne State University, Detroit, MI 48201, USA; swathi.srikantan@wayne.edu (S.S.); hp8688@wayne.edu (A.W.); 2 Hematology Oncology, Henry Ford Health, Detroit, MI 48236, USA; zmusleh2@hfhs.org; 3 Stem Cell Transplant and Cell Therapy, Henry Ford Health, Detroit, MI 48202, USA

**Keywords:** cytokine release syndrome, immune effector cell-associated neurotoxicity syndrome, immune effector cell-associated hemophagocytic lymphohistiocytosis-like syndrome, CAR-T cell therapy, anakinra, tocilizumab, predictive biomarkers, macrophage activation, neuroinflammation

## Abstract

Chimeric antigen receptor T (CAR-T) cell therapy has completely reshaped the therapeutic landscape for some blood cancers, including B-cell lymphoma, myeloma, and acute lymphoblastic leukemia. However, the complications associated with this powerful therapy are preventing it from being used more widely. This review explores the underlying biology, clinical signs, and current treatments for the three major early CAR-T cell therapy-associated toxicities: cytokine release syndrome, immune effector cell-associated neurotoxicity syndrome, and immune effector cell-associated hemophagocytic lymphohistiocytosis-like syndrome. Here, we outline key findings from preclinical animal models, data from multicenter clinical trials, and real-world patient cohort study data to delineate how infused cells and different host-derived and blood–brain barrier disruptions contribute to these adverse effects. A growing body of evidence is revealing that the patient’s own monocytes and macrophages, not just the infused CAR-T cells, may contribute to driving systemic toxicities primarily by releasing interleukin-1 (IL-1) and IL-6. A more thorough and mechanistically detailed understanding of CAR-T cell therapy-associated toxicity is needed to develop better predictive tools and treatment strategies so that patients derive the greatest antitumor benefits with the lowest risk of severe treatment-related complications.

## 1. Introduction

**Figure 1:**
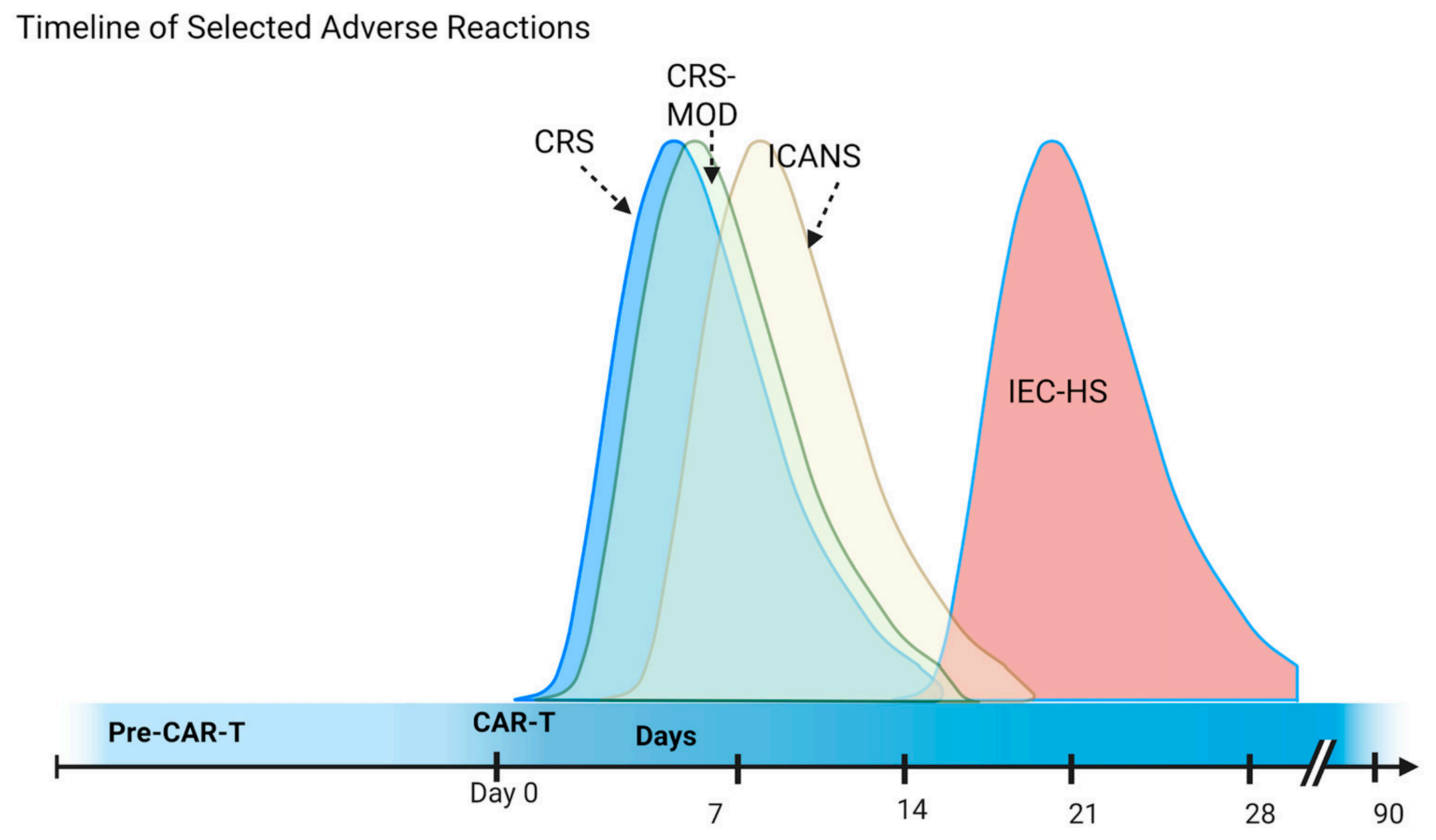
Early CAR-T cell-associated toxicity timings. CRS: cytokine release syndrome; CRS-MOD: cytokine release syndrome–multiorgan dysfunction; ICANS: immune effector cell-associated neurotoxicity syndrome; IEC-HS: immune effector cell-associated hemophagocytic lympho-histiocytosis-like syndrome. Created in *BioRender. Gangadaran, P. (2025)*. BioRender.com/g8k2s3b.

**Figure 2:**
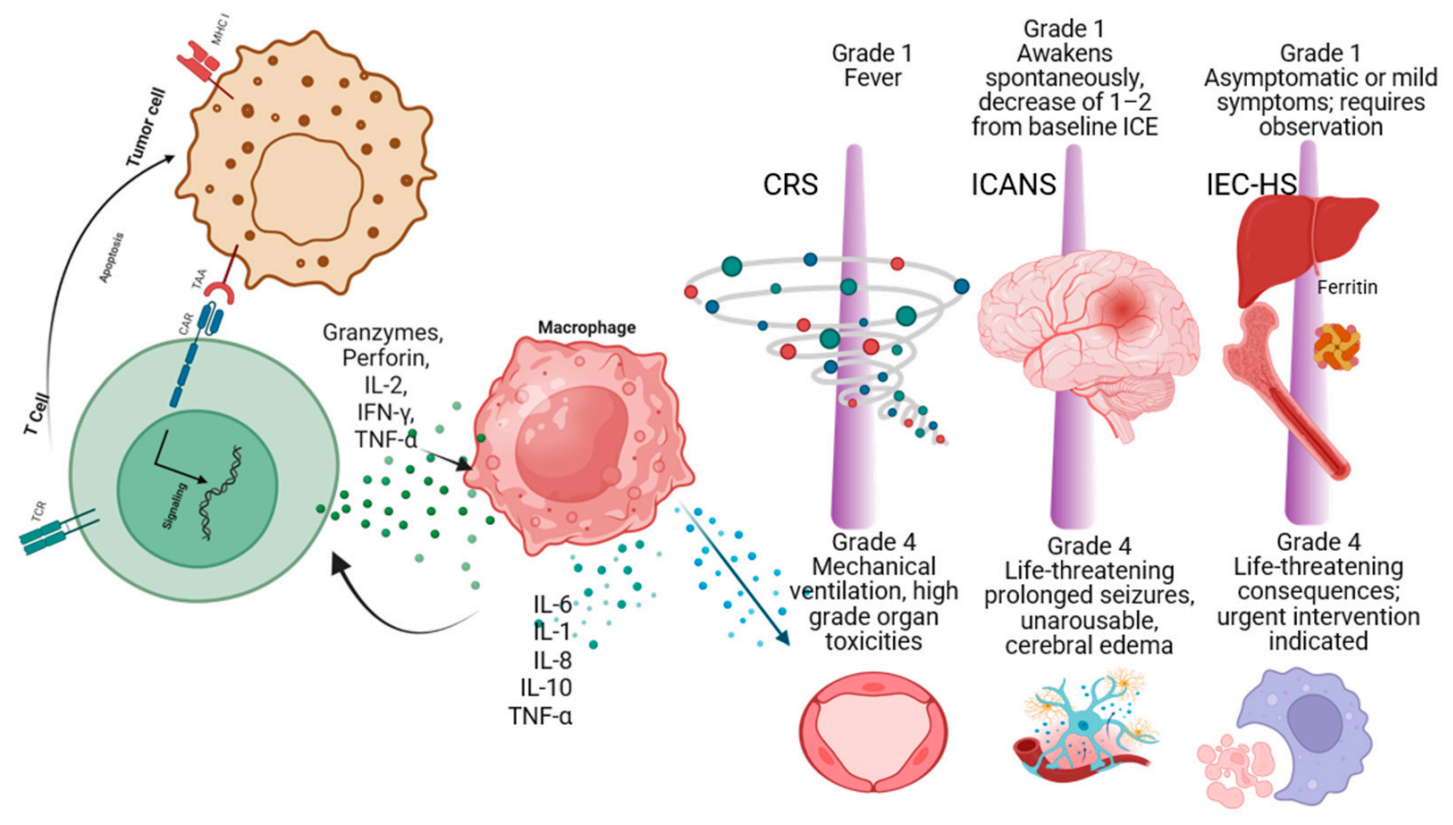
Early CAR-T cell-associated toxicities: mechanism and grading. CRS: cytokine release syndrome; ICANS: immune effector cell-associated neurotoxicity syndrome; ICE: immune effector cell encephalopathy; IEC-HS: immune effector cell-associated hemophagocytic lympho-histiocytosis-like syndrome; IFN: interferon; IL: interleukin; TNF: tumor necrosis factor. Created in *BioRender. Gangadaran, P. (2025)*. BioRender.com/g8k2s3b.

Chimeric antigen receptor T (CAR-T) cell therapy represents a breakthrough in modern cellular immunotherapy [1]. By modifying a patient’s own T cells to express synthetic receptors that target specific tumor antigens, including CD19 in B-cell leukemias and B-cell maturation antigen (BCMA) in multiple myeloma, the CAR-T approach successfully leverages the adaptive immune system, resulting long-lasting remission, even patients with advanced disease [2]. However, CAR-T cells can trigger several unique, life-threatening immune toxicities early after infusion (Figure 1) that are related to the therapeutic mechanism, mainly stemming from the massive in vivo expansion of engineered T cells that can result in cellular features that promote immune system hyperactivation (Figure 2). Of the three main CAR-T cell therapy-associated toxicities, the most common and well-known is cytokine release syndrome (CRS), a systemic inflammatory “cytokine storm” sparked by a massive release of cytokines from T cells binding to their target receptors. Next is immune effector cell-associated neurotoxicity syndrome (ICANS) [3], which can manifest as a wide range of effects, ranging from mild dysgraphia to life-threatening cerebral edema. And the most recently identified toxicity is immune effector cell-associated hemophagocytic lympho-histiocytosis-like syndrome (IEC-HS) [4], which is an independent hyperinflammatory condition characterized by macrophage activation leading to a severe drop in blood cell count and organ failure. Early clinical trials and institutions have varied widely in the assessment and grading of CAR-T cell therapy-associated toxicities, limiting comparisons of safety across various CAR constructs and hindering development of standardized approaches to toxicity management. To address this, the American Society for Transplantation and Cellular Therapy (ASTCT) brought experts together to standardize toxicity definitions and grading systems [3]. Today, this framework is universally used in clinical trials and everyday practice. In addition, a recent update was published to include additional toxicities that were recently recognized that are related to diseases, products, and class effects [5]. However, even with improved grading, there is still much to learn about how infused T cells interact with the innate immune system.

In this review, we examine the current models of the molecular mechanisms underlying early CAR-T cell-associated toxicities and various approaches to treating patients who develop CAR-T cell-associated toxicities. Through a close examination of CRS, ICANS, and IEC-HS, we highlight a major shift in our understanding of these toxicities as mediated not only by CAR-T cell-intrinsic features, but also by host immune cell responses. We also summarize strategies for predicting toxicity risk, including biomarker-based approaches, and emerging interventions that target pathways beyond those regulated by IL-6. Ultimately, a deeper understanding of the molecular basis of CAR-T cell therapy-associated toxicity is needed to facilitate advances in risk stratification and targeted management while still optimizing the therapy’s anti-cancer effectiveness.

## 2. Three of the Early CAR-T Cell Therapy-Associated Toxicities

### 2.1. Cytokine Release Syndrome

#### 2.1.1. Pathophysiological Mechanisms of CRS

CRS is the most common acute toxicity associated with CAR-T cell therapy, occurring in approximately 75% of patients [6], but its incidence can vary in the range of 50–100% and grades 3–4 can vary in the range of 1–30% [7]. This variation is related to many factors including CAR-T construct, costimulatory domain, target antigen, and disease burden. For example, CD28-based CAR-T cell products generally produce earlier and more intense CRS, whereas 4-1BB (CD137) co-stimulatory domain products are more often associated with delayed and less severe manifestations. CRS begins with antigen recognition and rapid CAR-T cell activation resulting in the release of inflammatory mediators, including interferon-γ (IFN-γ), tumor necrosis factor-α (TNF-α), and granulocyte-macrophage colony-stimulating factor (GM-CSF) [8]. However, accumulating evidence indicates that monocytes and macrophages, rather than the CAR-T cells themselves, are the principal downstream effectors of CRS (Figure 2) [9].

Therefore, our understanding of what drives CRS has recently been shifting. Preclinical studies have shown that macrophage-derived interleukin-1 (IL-1), IL-6, and nitric oxide contribute significantly to CRS severity, with macrophage depletion markedly attenuating the syndrome [10]. And the importance of myeloid cells in CRS was subsequently confirmed in a humanized mouse model, which identified monocytes as major sources of IL-1 and IL-6. Notably, IL-1β appeared to increase before IL-6, supporting a model in which IL-1 initiates the inflammatory cascade and IL-6 subsequently amplifies it [11].

IL-6 binds to both membrane-bound and soluble IL-6 receptors, and classic signaling primarily affects cells expressing membrane-bound receptors. However, IL-6 trans-signaling through soluble IL-6 receptor/gp130 complexes activates additional cell types, including endothelial cells, promoting vascular permeability, vasodilation, and further inflammatory signaling. Immune-mediated tumor-cell destruction may also contribute to the inflammatory response through cell-death pathways, such as pyroptosis, which releases damage-associated molecular patterns and enhances innate immune activation [11]. Endothelial injury is characterized by increased levels of angiopoietin-2 and the von Willebrand factor and increased expression of adhesion molecules such as ICAM-1 and VCAM-1, which contribute to capillary leak and coagulopathy in severe CRS [12]. Some researchers reported that the Endothelial Activation Stress Index (EASIX), calculated as lactate dehydrogenase (LDH, U/L) × creatinine (mg/dL) ÷ platelet count (10^9^ cells/L), serves as a possible surrogate marker of endothelial damage, while others reported that the modified EASIX (m-EASIX), which replaces creatine with C-reactive protein (CRP, mg/dL), has also shown value in predicting severe CRS post-CAR-T [12]. Emerging evidence also suggests that IFN-γ signaling may contribute to CRS-associated neutropenia through altered T-helper-cell differentiation [13].

#### 2.1.2. CRS Clinical Presentation and Severity Grading

The first several days following CAR-T cell infusion carry the highest risk of CRS, although timing and severity vary according to the CAR construct, disease burden, and degree of CAR-T cell expansion. CD28-based CAR-T cell products, such as axicabtagene ciloleucel, generally produce earlier and more intense CRS, whereas 4-1BB (CD137) co-stimulatory domain products, such as tisagenlecleucel and lisocabtagene maraleucel, are more often associated with delayed and less severe manifestations [14]. Although initial findings from recent clinical trials using dual-target CAR-T cell, like CD19/20 for B-cell lymphoma and CD19/BCMA for myeloma, show less severe CRS than single CAR therapies, this apparent safety advantage must be interpreted cautiously due to the extremely small sample sizes [15]. Further investigation is required to confirm these results, as the observed differences may also stem from variances in clinical practice across sites and the modern implementation of pre-emptive therapy for early toxicity management.

The clinical spectrum of CRS is wide, extending from isolated fever to profound hypoxemia, and multiorgan dysfunction (Figure 2). Early CRS typically occurs within 72 h of infusion and is particularly associated with high tumor burden and rapid CAR-T cell expansion. Delayed CRS, on the other hand, occurs later, sometimes 5 days after infusion, and warrants continued monitoring, particularly with 4-1BB-based products. Severe inflammation characterized by high ferritin, low fibrinogen, liver dysfunction, and coagulopathy can resemble hemophagocytic lymphohistiocytosis. When this clinical phenotype develops during CRS, it is usually referred to as CRS with multiorgan dysfunction. However, when it occurs independently from CRS and ICANS, it is classified separately as IEC-HS [5] (Section 2.3).

The ASTCT consensus criteria define CRS by the presence of fever, with severity graded based on the development of hypotension, hypoxia, and other complications [3,5]. Importantly, organ dysfunction associated with CRS is graded independently with the Common Terminology Criteria for Adverse Events (CTCAE) and this feature is not incorporated into the CRS grade.

Cardiovascular complications are an important feature of severe CRS that may contribute substantially to morbidity and mortality [16,17]. The underlying CRS-associated cardiac phenotype resembles sepsis-associated myocardial dysfunction with systemic inflammation and impaired myocardial contractility mediated by JAK-STAT3-mediated IL-6 signaling [18]. Troponin elevation has been reported in a high proportion of patients with grade ≥ 2 CRS, and a transient reduction in left ventricular ejection fraction (LVEF) that generally improves as CRS resolves is seen in approximately 5% to 12% of patients. At our institution, we have seen that most cardiac events occur within the first 6 months after infusion, while late-onset complications are infrequent [19].

The cardiovascular manifestations of CAR-T cell therapy may also include sinus tachycardia, atrial fibrillation and other tachyarrhythmias, QTc prolongation, myocardial injury, and systolic dysfunction. Severe CRS may progress to cardiogenic or mixed distributive cardiogenic shock. Importantly, cardiovascular events do not always parallel CRS severity and may also arise in the absence of clinically significant CRS, suggesting contributions from lymphodepleting chemotherapy, systemic inflammation, or other immune-mediated mechanisms.

Patients with moderate-to-severe CRS should therefore undergo close cardiovascular monitoring with baseline echocardiography assessment, which is particularly essential for patients with pre-existing cardiovascular disease. For patients with grade ≥ 2 CRS, continuous cardiac monitoring, serial electrocardiography, assessment of cardiac biomarkers, and echocardiographic evaluation can facilitate early recognition of myocardial injury and hemodynamic deterioration [20]. Treatment is primarily directed toward controlling the underlying CRS while providing standard supportive treatment for arrhythmias, ventricular dysfunction, and shock. Prompt recognition of cardiac involvement is particularly critical because hypotension caused by CRS-related vasodilation may coexist with impaired ventricular function, requiring careful fluid and vasopressor management.

#### 2.1.3. Biomarkers and Risk Prediction

The risk and severity of CRS are influenced by baseline disease characteristics and the specific CAR-T cell construct. High tumor burden, rapid CAR-T cell expansion, and elevated inflammatory markers, including C-reactive protein, ferritin, and lactate dehydrogenase, have consistently been associated with more severe CRS [21]. Cytokines such as IL-6, IL-10, and TNF-α have also been investigated as potential predictive biomarkers [22] (Table 1).

However, composite models may provide greater predictive value than individual biomarkers. Distinguishing CRS from sepsis in patients undergoing CAR-T cell therapy is an important diagnostic challenge, and various models have been investigated [26]. But while these models may improve risk stratification, their applicability across different CAR-T cell products and disease settings requires further validation.

#### 2.1.4. CRS Therapeutic Approaches

Choosing an appropriate CRS treatment is primarily guided by ASTCT grading and institutional or National Comprehensive Cancer Network (NCCN) recommendations. Because CRS may resemble sepsis, particularly in patients with neutropenia, infectious causes should be promptly investigated and empiric broad-spectrum antibiotics initiated when clinically appropriate.

Patients with grade 1 CRS are generally treated with supportive measures, such as antipyretic and fluid administration, as well as close observation. In some patients with early-onset CRS, recent recommendations suggest considering early tocilizumab injection and/or steroids [27]. However, grade 2 CRS generally warrants intravenous (IV) tocilizumab at 8 mg/kg (maximum 800 mg), with up to 4 repeat doses at 8 h intervals when necessary. Dexamethasone and cautious IV fluid administration may be added, particularly when symptoms persist, or hypotension or hypoxia develops [27]. Notably, patients with grade 3 CRS require intensive monitoring, often in an ICU setting, with vasopressor support for persistent hypotension and appropriate oxygen or ventilatory support. In this situation, tocilizumab may be combined with corticosteroids, such as dexamethasone, with treatment intensity determined by the clinical trajectory [27]. Grade 4 CRS requires treatment in a critical care setting and may involve multiple vasopressors and positive-pressure ventilation. Patients with severe refractory CRS may require high-dose corticosteroids, such as methylprednisolone, in addition to IL-6 blockade [27], and refractory CRS unresponsive to corticosteroids and tocilizumab may require alternative immunomodulatory strategies. For instance, the IL-1 receptor antagonist anakinra is increasingly favored as a third-line option because IL-1 signaling appears to occur upstream of IL-6 in CRS pathogenesis [11]. Other approaches for refractory disease, including siltuximab and clazakizumab, which are monoclonal antibodies that bind to IL-6, have also been explored, although supporting clinical evidence remains limited [27].

### 2.2. Immune Effector Cell-Associated Neurotoxicity Syndrome

#### 2.2.1. Pathophysiological Mechanisms of ICANS

ICANS is an acute neurologic toxicity with a multifaceted pathophysiology that may involve interactions between systemic immune activation, myeloid-cell signaling, neurovascular unit disruption, and inflammation of the central nervous system (CNS) (Figure 2). It can occur regardless of the presence of CNS involvement by the tumor. In a humanized mouse model of high leukemia burden, CAR-T cell-mediated tumor clearance led to severe neurotoxicity and an inflammatory response resulting in the activation of endogenous and or infused T cells and/or other immune effector cells [5], with IL-1 and IL-6 mainly produced by human monocytes [10]. In this model, depleting monocytes and blocking IL-6 receptor signaling with tocilizumab both prevented CRS; however, tocilizumab did not protect against delayed, lethal neurotoxicity associated with meningeal inflammation. Notably, blockade of IL-1 signaling prevented both CRS and neurotoxicity, resulting in longer leukemia-free survival [10]. These data implicate monocyte-derived IL-1 as a key driver of CAR-T cell-associated neurologic toxicity and suggest that suppressing IL-6-mediated systemic inflammation alone may not prevent CNS injury.

Inflammatory signaling may be accompanied by immune cell trafficking into the CNS. In a rhesus macaque model, autologous CD20-directed CAR-T cells induced neurotoxicity and CRS during CAR-T cell expansion [28]. The neurotoxicity was associated with an increase in proinflammatory cytokines in cerebrospinal fluid and the accumulation of both CAR and non-CAR-T cells in the cerebrospinal fluid and brain parenchyma. Histopathologic examination revealed perivascular T-cell infiltration and pan-T-cell encephalitis [28]. Although this model used CD20-directed CAR-T cells, the results support a mechanism in which compartmentalized cytokine production and recruitment of multiple T-cell populations can contribute to inflammation within the CNS.

Endothelial activation and blood–brain barrier (BBB) disruption may provide a mechanistic link between systemic inflammation and central neurotoxicity. In a study including 133 patients treated with CD19-directed CAR-T cells, severe neurotoxicity was associated with capillary leak, coagulopathy, endothelial activation, and increased BBB permeability [14]. Loss of barrier integrity exposed the cerebrospinal fluid to high concentrations of circulating cytokines, including interferon-γ, inducing stress in brain vascular pericytes and stimulating the release of endothelial-activating mediators [14]. Neuropathologic examination of a patient who had fatal neurotoxicity showed multifocal vascular injury and perivascular CD8-positive T-cell infiltration [14]. Although these observations support a central role for neurovascular dysfunction in ICANS, they do not establish endothelial injury as independently sufficient cause.

An antigen-specific mechanism of CAR-T cell-associated neurovascular injury has also been proposed. Mural cells, including pericytes, support vascular stability and BBB integrity, and single-cell RNA sequencing has shown that these cells express CD19 [29]. Therefore, CD19-directed CAR-T cell recognition of these cells could contribute to on-target, off-tumor toxicity. However, the study did not show direct CAR-T cell-mediated injury to human mural cells, but the authors suggested that lower CD19 expression in murine mural cells may limit the utility of conventional mouse models for evaluating this mechanism [29]. In addition, on a microscopic examination, some show changes that are consistent with blood–brain barrier dysfunction, like enlarged perivascular spaces with fluid extravasation. Polymerase chain reaction was used to detect the presence of CAR-T cells [30]. Thus, CAR-T cells targeting mural cells remain a biologically plausible but unconfirmed mediator of ICANS.

CAR-T cell-mediated cerebral microvascular dysfunction may further disrupt the BBB and neurovascular function. In an immunocompetent mouse model of CD19-directed CAR-T cell therapy, neurotoxicity was accompanied by BBB disruption, microhemorrhages, reduced pericyte coverage, and capillary obstruction associated with patchy cerebral hypoxia and substantial behavioral impairment [31]. Capillary obstruction was mediated by CD45-positive leukocytes, including CD3-positive T cells [31]. These data suggest that increased leukocyte–endothelial adhesion and reduced cerebral microvascular perfusion may link systemic immune activation to neurologic dysfunction, although the contribution of this mechanism to human ICANS has not been established.

In CAR-T cell-associated neurotoxicity, resident CNS immune cells may amplify injury following neurovascular disruption. In B-cell lymphoma-bearing mice, CD19-directed CAR-T cell transfer activated the transforming growth factor-β-activated kinase 1 (TAK1)-nuclear factor-κB-p38 mitogen-activated protein kinase pathway, inducing microglial activation and neurocognitive impairment [32]. But notably, genetic and pharmacologic inhibition of microglial TAK1 reduced neuroinflammation and improved neurocognitive performance without eliminating CAR-T cell antitumor function [32]. In human patients with ICANS, translocator protein positron emission tomography has revealed microglial activation, and imaging mass cytometry has shown a shift from resting toward activated microglial states in human CNS tissue [32]. Severe ICANS may also involve direct glial and white matter injury, as demonstrated by postmortem findings of multifocal demyelinating leukoencephalopathy and region-specific loss of oligodendroglial lineage cells in a patient with grade 4 ICANS after CD19-directed CAR-T cell therapy [33]. These findings, although limited to a single patient, suggested a potential cellular correlate of severe ICANS [33].

Together, current evidence supports a model of ICANS in which CAR-T cell activation initiates myeloid cell cytokine release, endothelial activation, BBB disruption, and immune cell entry into the CNS. Cerebral microvascular impairment and microglial activation may subsequently amplify neuroinflammation, while oligodendroglial and white matter injury may occur in severe disease. However, the temporal sequence and relative contribution of these mechanisms in patients with ICANS remain incompletely defined.

#### 2.2.2. ICANS Clinical Phenotypes and Severity Grading

ICANS encompasses a heterogeneous spectrum of neurologic manifestations and is best described according to its dominant clinical features, temporal course, and severity rather than by discrete subtypes. The American Society for Transplantation and Cellular Therapy grades ICANS across five domains: an immune effector cell-associated encephalopathy (ICE) score, level of consciousness, seizure activity, motor abnormalities, and evidence of elevated intracranial pressure or cerebral edema (Figure 2) [3]. The ICE score evaluates orientation, naming, command following, writing, and attention, and the overall ICANS grade is determined by the most severe abnormality within any domain that cannot be attributed to another cause [3]. Therefore, the ICE score is a measure of encephalopathy rather than a complete assessment of the neurologic syndrome. Recently, the ASTCT Toxicity Consensus Group endorsed the ICE tool for adults and approved its use for children aged 9 years and older, while introducing the modified ICE to be used for children or patients with developmental delays for whom ICE scoring is not appropriate [5].

ICANS typically arises shortly after CAR-T cell infusion, although its onset and duration vary. In a multicenter study of 84 patients treated with CD19-directed CAR-T cells for relapsed lymphoma, neurologic toxicity developed in 43% at a median of 7 days after infusion, persisting for a median of 6 days [34]. Among affected patients, 64% developed grade 1 or 2 toxicity, whereas 36% developed grade 3 or 4 toxicity [34]. In a prospective single-center study of 53 patients who received CAR-T cell therapy for hematological malignancies, 12 developed neurotoxicity, including 10 with grade 1 diseases. Changes in handwriting and tremors were the initial manifestations in all 12 patients, and neurologic symptoms resolved within one month [35]. These studies show that the early clinical course of ICANS may begin with subtle changes before progressing to more overt encephalopathy.

Cognitive and language disturbances are among the most characteristic manifestations of ICANS. In a series of 100 CAR-T cell recipients, the most frequently documented neurologic symptoms were encephalopathy, headache, tremor, aphasia, and focal weakness [36]. While language dysfunction may include anomia, reduced verbal output, impaired fluency, dysgraphia, or global aphasia, the cognitive syndrome often extends beyond language impairment, with one study observing executive dysfunction, cognitive slowing, apraxia, disorientation, impaired attention, dyscalculia, hemispatial neglect, restlessness, hallucinations, and confusional states in CAR-T cell recipients [34]. While approximately half of the patients with cognitive impairment in that group developed language dysfunction, the remainder had other cognitive deficits [34]. These observations indicate that although aphasia is a characteristic feature of ICANS, it does not account for the entire encephalopathic syndrome.

Motor manifestations in ICANS may include tremors, myoclonus, asterixis, dyskinesia, cerebellar dysfunction, and focal weakness [34,36]. Nonpyramidal motor abnormalities, including postural tremors, myoclonus, asterixis, upper-extremity dyskinesia, and static or kinetic cerebellar abnormalities, were observed in 42% of patients in one study [34]. However, several of these manifestations are not directly incorporated into ASTCT grading unless they are accompanied by encephalopathy or a severe focal motor deficit. Consequently, clinically apparent movement abnormalities may not be fully represented by the ICE score or overall ICANS grade.

Seizures are less common than encephalopathy in patients with ICANS, but seizure indicates a more severe neurologic involvement, which may be focal or generalized and can occur without prominent convulsive activity. In the study by Belin et al., three patients developed generalized tonic–clonic seizures [34]. And in another study, 28 of 56 patients undergoing CAR-T cell therapy developed ICANS, with electroencephalography (EEG) showing abnormalities in 89% of those evaluated after symptom onset. Encephalopathic EEG patterns were present in 70% of patients, while interictal epileptiform discharges occurred in 52%, which were associated with progression to a higher ICANS grade within 24 h, although this finding requires validation [37].

Patients with severe ICANS may present with profound encephalopathy, depressed consciousness, prolonged or repetitive seizures, focal motor deficits, or cerebral edema. Under the ASTCT system, grade 3 or 4 toxicity may be assigned on the basis of severe ICE-score impairment, reduced arousability, seizure activity, major focal weakness, or radiographic or clinical evidence of cerebral edema [3]. Structural neuroimaging may remain normal despite substantial neurologic dysfunction. Focal neurologic deficits have been reported to more consistently correlate with regional EEG abnormalities, cerebral hypometabolism on fluorodeoxyglucose positron emission tomography, and elevated transcranial Doppler velocities than with abnormalities on structural imaging [36].

Although ICANS is usually acute and monophasic, delayed or relapsing presentations can occur. In one report, a patient developed grade 4 ICANS with coma 14 days after CAR-T cell infusion, recovered, and experienced a second episode on day 40 with disorientation, confabulation, and focal seizures [38]. Although this example highlights the possibility of delayed relapse, it does not establish a distinct biphasic clinical subtype [38]. Overall, ICANS ranges from subtle changes in handwriting, attention, language, and motor control to seizures, profound encephalopathy, coma, and cerebral edema. Importantly, the ICE score may not capture the full spectrum of neurologic effects associated with CAR-T cell therapy, highlighting the need for broader neurologic assessment.

#### 2.2.3. Biomarkers of ICANS

Biomarkers that have been explored for ICANS reflect systemic inflammation, endothelial and BBB dysfunction, neuroaxonal or astroglial injury, and cerebral electrical activity. However, interpretation of biomarkers for ICANS is challenging due to differences in CAR-T cell products, underlying malignancies, specimen types, sampling time, and ICANS grading. Several proposed markers may also be altered during CRS or may reflect pre-existing neurologic injury rather than ICANS-specific pathology.

Inflammatory cytokines and markers of BBB disruption have been associated with severe neurotoxicity. In one study of 53 adults with B-cell acute lymphoblastic leukemia treated with CD19-directed CAR-T cells, severe neurotoxicity was accompanied by greater CAR-T cell expansion and higher circulating concentrations of several proinflammatory cytokines [39]. Cerebrospinal fluid protein concentrations correlated with neurotoxicity grade, whereas IL-6, IL-8, monocyte chemoattractant protein-1, and interferon-γ-induced protein 10 were elevated in the cerebrospinal fluid during severe neurotoxicity [39]. In another study of 133 patients, severe neurotoxicity was associated with endothelial activation, coagulopathy, and increased BBB permeability, with lower angiopoietin-1 concentrations and higher angiopoietin-2-to-angiopoietin-1 ratios indicating reduced vascular stability [14]. These findings support the inflammatory and neurovascular basis of ICANS, although overlapping with CRS limits the diagnostic specificity of these biomarkers.

The CXCL16/CXCR6 axis has emerged as a potential marker of CNS inflammation. A study comparing peripheral blood and cerebrospinal fluid samples from 11 patients who developed ICANS after CAR-T cell therapy showed that CXCR6-positive cytotoxic CD4 CAR-T cells were enriched in the cerebrospinal fluid, and CXCL16 concentrations in the cerebrospinal fluid correlated with ICANS grade [40]. The study also observed a spatial association between CXCR6-positive T cells and CXCL16-expressing myeloid cells in postmortem tissue from a patient with fatal grade 4 ICANS. However, this study was small, and CAR-T cell recipients without ICANS were not included as control patients. Therefore, the diagnostic specificity and predictive value of CXCL16 remain undetermined.

Markers of neuroaxonal and astroglial injury may more directly reflect CNS involvement in ICANS. In a study of 96 patients with refractory B-cell malignancies who developed ICANS, serum neurofilament light-chain (NfL) concentrations increased after CAR-T cell infusion, and higher concentrations were associated with greater disease severity [41]. Pretreatment elevated levels of NfL were also associated with subsequent toxicity, suggesting that serum NfL may reflect both baseline neuroaxonal vulnerability and treatment-related injury [41]. In a study of pediatric and young adult patients with hematological malignancies who received either CD19- or CD19/CD22-directed CAR-T cell therapy, cerebrospinal fluid glial fibrillary acidic protein (GFAP) and NfL [23] increased in those who developed ICANS and had CD19-directed therapy, and increases were more prominent in those with more severe neurotoxicity, supporting a model of concurrent astroglial and neuroaxonal injury in ICANS; however, why patients who received CD19/CD22 CAR-T cell therapy did not show an equivalent increase in GFAP and NfL remains unknown. Overall, biomarkers that indicate direct CNS injury in ICANS may differ depending on the sampled compartment, patient population, and CAR-T cell construct. In addition, different reports used different measuring assays that are similar in performance but differ in sensitivity.

S100 proteins have also been evaluated as markers of BBB and glial injury. In a small retrospective study, serum S100 concentrations increased in patients who developed ICANS, and persistent elevation was associated with greater neurologic severity [24]. However, an observational study of 72 patients who received CD19-based CAR-T cell therapy observed that, although the 21 patients who developed ICANS had higher levels of early serum S100β, the only significant predictor of ICANS in the study was previous CNS involvement [42]. Therefore, S100β may reflect underlying CNS vulnerability or nonspecific BBB stress rather than being an ICANS-specific injury marker.

EEG has been investigated as a noninvasive measure of cerebral dysfunction in ICANS. In a retrospective study of 120 CAR-T cell recipients who underwent EEG monitoring, the authors proposed a machine learning-based visual EEG-ICANS scoring system of visually assessable disease-associated EEG features. The scoring system significantly correlated with ICANS severity and discriminated against grade 2-and-higher ICANS [43]. However, although pre-infusion EEG patterns have been investigated for predicting ICANS risk and severity, the proposed scoring system assesses concurrent cerebral dysfunction only, and external validation of the system is needed.

In summary, no single biomarker for ICANS that combines sufficient specificity, reproducibility, and clinical accessibility is available to replace serial neurologic assessment (Table 1). While inflammatory and endothelial markers can characterize systemic and neurovascular activation, cerebrospinal fluid GFAP, NfL, and CXCL16 may provide more direct evidence of CNS involvement. Serum S100β remains insufficiently specific for ICANS, and EEG principally measures active cerebral dysfunction. Future studies should evaluate multimarker approaches incorporating standardized sampling intervals, uniform ASTCT grading, and independent validation across CAR-T cell products and disease populations.

#### 2.2.4. Toxicity Prediction

Early identification of patients at risk for ICANS remains difficult because no clinical variable, biomarker, or multivariable model has been validated across CAR-T cell products and disease-specific populations. Reported risk indicators include both disease- and treatment-related factors, such as high disease burden, thrombocytopenia, and CAR-T cell product, as well as early or severe CRS, greater CAR-T cell expansion, and early inflammatory activation [14,39,44]. Pre-infusion elevated serum NfL may identify patients with reduced baseline neuroaxonal integrity, although the findings have been inconsistent across adult and pediatric cohorts [23]. Also, although abnormal pre-infusion EEG patterns have been associated with subsequent severe ICANS, the value of this approach as a screening measure remains uncertain [37].

A study of 101 patients treated with axicabtagene ciloleucel or tisagenlecleucel aimed to develop a multivariable risk model of CD19 CAR-T cell therapy-associated ICANS by identifying risk factors and disease-specific cytokine expression patterns [44]. Two models that each showed good, but not excellent, discriminatory power for ICANS were developed. The first model incorporated the CAR-T cell product, time to CRS onset, plasma IL-6 concentration on day 3, and pre-infusion D-dimer levels for predicting any-grade ICANS. The second model was for predicting grade 2-to-4 ICANS and included the number of previous therapies, presence of grade 2-or-greater CRS, history of autoimmune disease, pre-infusion IL-15 levels, and granulocyte–macrophage colony-stimulating factor [44]. But notably, the models were developed based on retrospective data and were not tested in an external population; therefore, changes in treatment protocols during the study period may have influenced the results, and the models need to be verified.

Current evidence indicates that disease and patient characteristics, CAR-T cell product, and magnitude and timing of the early inflammatory response may collectively help predict ICANS risk. Multivariable approaches may be more robust than isolated laboratory measurements, but existing models remain exploratory. External validation, prospective collection of biomarkers at standardized intervals, and assessment across different CAR-T cell constructs and malignancies are required before risk estimates can guide prophylaxis or alter routine monitoring.

#### 2.2.5. ICANS Therapeutic Approaches

Therapeutic approaches for ICANS depend on early recognition, exclusion of competing diagnoses, and treatment selection according to the most severe neurologic manifestation. Serial ICE scoring and neurologic examinations should be performed during the period of greatest post-infusion risk, with closer monitoring after any decline in attention, language, handwriting, orientation, or level of consciousness [3,45]. Evaluation should also include a medication review, identification of metabolic disturbances, and infectious disease assessment. EEG is indicated when seizures, nonconvulsive status epilepticus, or unexplained encephalopathy are suspected [37,43,45]. Brain magnetic resonance imaging (MRI) and cerebrospinal fluid analysis are generally not used to confirm ICANS, but rather, are helpful to exclude stroke, infection, malignant involvement, and other neurologic disorders [45]. In one study of children and young adults with acute ICANS, MRI most often showed abnormalities in the cerebral white matter, brainstem, and thalami, particularly with higher-grade ICANS [46].

Grade 1 ICANS is generally managed with close observation and supportive care. Corticosteroids are the primary treatment for grade 2-or-greater ICANS, while tocilizumab should be reserved for patients with concurrent CRS because it has limited activity against isolated neurotoxicity [45]. Suggested regimens for grade 2-or-3 ICANS include 10 to 20 mg IV dexamethasone every 6 h, followed by a rapid taper after neurologic improvement. Grade 4 ICANS warrants intensive care and IV methylprednisolone 1 g daily for at least 3 days, with subsequent treatment determined by the clinical response [45]. Seizures are commonly treated with levetiracetam, while benzodiazepines are used for status epilepticus. Routine antiseizure prophylaxis in patients without seizures is not recommended by the EBMT/EHA guidance, although institutional practices vary [45].

The role of prophylactic corticosteroids remains uncertain. Cohort 6 of the ZUMA-1 CAR-T cell therapy clinical trial was a safety management cohort for exploring the role of prophylactic corticosteroids and tocilizumab intervention for CRS and neurologic complications. Of 40 patients with large B-cell lymphoma who received axicabtagene ciloleucel with oral dexamethasone and preventive corticosteroid or tocilizumab intervention for subsequent toxicity, CRS occurred in 80% of patients, with no grade 3-or-higher events [47]. However, 58% developed neurologic events, with 13% of those being grade 3 or higher [47]. Although the antitumor responses were maintained, the nonrandomized design and concurrent use of earlier therapeutic intervention make it difficult to determine the independent effect of prophylactic therapy on ICANS incidence and severity.

Anakinra is an immunomodulatory agent increasingly used ICANS that progresses despite high-dose corticosteroids, and its use is supported by experimental evidence implicating monocyte-derived IL-1 in CAR-T cell-associated neurotoxicity [10]. In a study of 14 patients with steroid-refractory ICANS, ICANS or refractory CRS grade decreased in 9 patients within 1 day after the final anakinra dose; however, doses were relatively low, and all patients received additional immunosuppressive and supportive therapies [28]. And a retrospective study of 43 patients treated with anakinra for refractory ICANS or CRS showed that doses up to 12 mg/kg/day were feasible, with a CAR-T cell objective response rate of 77% [48]. And while higher doses were associated with a lower rate of treatment-related mortality, the findings were limited by variations in dosing, baseline severity, concurrent corticosteroid exposure, and the absence of a control group [48]. Overall, these studies support anakinra as a reasonable option for steroid-refractory ICANS, but the optimal dose, timing, and duration remain unclear, as does its efficacy relative to other therapies.

In patients with progressive or refractory ICANS, reassessment should include consideration of infection, metabolic encephalopathy, stroke, or malignant CNS involvement, along with a review of corticosteroid dosing [49]. Care in this setting requires a multidisciplinary approach involving cellular therapy, neurology, critical care, and infectious disease specialists. Patients with cerebral edema require neurocritical care, intracranial pressure management, and close respiratory and hemodynamic monitoring. Intrathecal administration of corticosteroids or chemotherapy has been used to deliver immunosuppressive agents directly into the cerebrospinal fluid, although evidence is limited to small series and case reports [49]. An alternative approach is to target the CAR-T cells themselves, although these strategies are not well-established. Pharmacologic suppression is an approach used to temporarily reduce CAR-T cell signaling or activity, whereas a depletion approach eliminates infused cells through cytotoxic chemotherapy or activation of an engineered suicide switch. However, tunable CAR-T cell constructs, and suicide-switch systems must be incorporated into the product design, and there are not available in most commercial products [45,49]. And because CAR-T cell suppression or depletion may also compromise antitumor activity, these approaches have generally been explored for severe, life-threatening toxicity unresponsive to conventional treatment.

Treatment decisions for ICANS currently rely on serial clinical assessment rather than biomarker-directed intervention, and prompt grading, exclusion of alternative diagnoses, corticosteroid treatment, seizure control, and early intensive care remain central. And although anakinra is an option for steroid-refractory disease, intrathecal treatment and direct suppression or depletion of CAR-T cells are still exploratory salvage strategies.

### 2.3. Immune Effector Cell-Associated Hemophagocytic Lymphohistiocytosis-like Syndrome

#### 2.3.1. Pathophysiological Mechanisms of IEC-HS

IEC-HS is an intense inflammatory response caused by CAR-T cells that is marked by new or worsening low blood cell counts, elevated ferritin, elevated liver enzymes, or clotting disorders involving low fibrinogen [5]. Although the pathophysiology of IEC-HS is not fully understood, it is thought to result from a sustained, unresolved hyperinflammatory response following CAR-T cell therapy-associated immune effector cell activation. Although IEC-HS shares many clinical and laboratory features of primary hemophagocytic lymphohistiocytosis (HLH), it does not fully meet the diagnostic criteria for HLH, and it is specific to the setting of immune effector cell therapy. Following antigen recognition, CAR-T cells undergo expansion, cytotoxic degranulation, tumor-cell lysis, and cytokine release, which recruits and activates myeloid cells. Activated monocytes, in turn, produce IL-1 and IL-6, amplifying the inflammatory response implicated in IEC-HS [10]. The ASTCT IEC-HS working group [3] and, recently, ASTCT Toxicity Consensus Group [5] stressed that it presents either as CRS that is actively resolving or following its complete resolution (Figure 1), as it is related to CAR-T cell activation that promotes continued T-cell proliferation and cytokine release, followed by activation of macrophages and other endogenous immune cells. Reciprocal T-cell–macrophage activation is associated with increased concentrations of IFN-γ, IL-1β, IL-6, IL-10, IL-12, IL-18, TNF-α, soluble IL-2 receptor, and ferritin [4]. However, the mechanisms driving the persistent inflammatory response after CRS subsides and the progression to IEC-HS are not completely understood.

The proposed mechanism of IEC-HS draws partly from HLH, in which impaired lymphocyte cytotoxicity prevents effective termination of the inflammatory immune response. In a perforin-deficient murine model of HLH, disease depended on persistent CD8-positive T-cell activity and IFN-γ signaling, supporting a model in which ineffective cytotoxic regulation allows continued T-cell stimulation, with subsequent macrophage activation and sustained cytokine production [50]. In IEC-HS, prolonged CAR-T cell signaling may produce dysregulated T-cell–macrophage interactions similar to those seen in HLH, even in the absence of genetic defects in granule-mediated cytotoxicity [4,50].

Support for this mechanism comes from a syngeneic model of anti-CD19 CAR-T cell therapy, in which perforin-deficient CAR-T cells showed reduced cytotoxicity while retaining the ability to eliminate leukemic cells. Following CAR activation, these cells produced more proinflammatory cytokines and re-expanded after tumor clearance, leading to splenomegaly, disruption of splenic architecture, and hemophagocytosis [51]. A similar pattern was observed in patients who developed HLH-like inflammation after CD22-directed CAR-T cell therapy, with inflammation occurring in two distinct phases accompanied by late CAR-T cell expansion and elevated IL-1β and IL-18 [33]. These data suggest that ineffective cytotoxicity and persistent or recurrent CAR-T cell expansion can sustain inflammation even after the target-cell burden has declined. However, whether this mechanism contributes to IEC-HS associated with currently available CAR-T cell products is unclear.

Studies of cytokine profiles in patients suggest that HLH-like toxicity involves a distinct inflammatory response rather than simply a more pronounced form of the cytokine response seen in severe CRS. Among 99 patients treated with B-cell maturation antigen-directed CAR-T cells, 20 developed study-defined CAR-T cell-associated HLH-like toxicity [52], with the active HLH-like phase characterized by greater elevations in IFN-γ, granzyme B, IL-1 receptor antagonist, and IL-10 than those seen in severe CRS [52]. IL-6 levels distinguished mild from severe inflammation but did not distinguish severe CRS from HLH-like toxicity. Together, these findings implicate sustained cytotoxic lymphocyte activation, IFN-γ signaling, macrophage stimulation, and counter-regulatory cytokine responses in IEC-HS but do not establish which mediators initiate or sustain the inflammatory response.

The timing and pattern of HLH-like inflammation after CD22-directed CAR-T cell therapy also suggests that inflammation may arise through more than one mechanism. A CRS-associated phenotype marked by extreme hyperferritinemia, coagulopathy, hepatic injury, cytopenias, and other manifestations of HLH-like inflammation has been observed [53]. In this phenotype, all affected patients first developed CRS, indicating that HLH-like hyperinflammation arose in the initial inflammatory response. In contrast, a second phenotype arose after CRS resolution and was accompanied by renewed CAR-T cell expansion [51]. Together, these patterns suggest that this manifestation may arise through different mechanisms, including through continued escalation of inflammation during active CRS or persistence or reactivation of T-cell–macrophage-associated inflammation after CRS has resolved.

Collectively, available evidence supports a model of IEC-HS in which CAR-T cell activation and tumor-cell lysis initiate inflammatory signaling followed by recruitment and activation of monocytes and macrophages. Persistent antigenic stimulation, ineffective termination of cytotoxicity, or recurrent CAR-T cell expansion may then sustain inflammation, with IFN-γ, IL-1-family cytokines, and related mediators reinforcing reciprocal activation between lymphoid and myeloid cells. This sustained hyperinflammatory response is associated with hyperferritinemia, progressive cytopenias, hypofibrinogenemia, hepatic injury, and multiorgan dysfunction. However, the initiating mechanism may differ between CRS-associated multiorgan dysfunction and delayed IEC-HS, but the relative contribution of individual cytokines has not been established.

#### 2.3.2. Clinical CRS-Associated Phenotypes and Related HLH-like Syndromes

HLH-like toxicities following CAR-T cell therapy are clinically and biochemically similar to primary HLH but are defined by their occurrence within the context of immune effector cell therapy. The ASTCT established the IEC-HS terminology to differentiate the pathologic and biochemical hyperinflammatory syndrome attributable to immune effector cell therapy that develops independently of CRS and ICANS. IEC-HS specifically involves macrophage activation and is clinically characterized by hyperferritinemia, new or progressive cytopenias, coagulopathy with hypofibrinogenemia, elevated hepatic transaminase, and possible multiorgan dysfunction [4]. Hemophagocytosis may be present in bone marrow or other tissues, but this feature is not required for diagnosis. The HLH-2004 criteria and H Score were developed for primary HLH and may be less applicable after CAR-T cell therapy because diagnostic features such as cytopenias and fever may be present at baseline or arise from the underlying malignancy or other post-infusion toxicities [4].

Two clinically relevant CAR-T cell-associated HLH-like patterns have been described: IEC-HS and CRS with multiorgan dysfunction (CRS-MOD) (Figure 1) [54]. CRS-MOD develops within the setting of active or worsening CRS, with progressive organ dysfunction and HLH-like biochemical abnormalities despite standard CRS-directed therapy. CRS-MOD is distinguished from transient CRS-associated hyperferritinemia, cytopenias, and organ injury by progressive dysfunction despite conventional treatment. In contrast, IEC-HS is clinically and temporally distinct from active CRS, typically emerging as CRS is improving or after it has resolved [4,5,54]. Therefore, the distinction relies on the clinical course in relationship to CRS rather than a single laboratory threshold. Differentiating CRS-MOD from IEC-HS is difficult within the context of concomitant CRS, infection, or other causes of organ dysfunction.

Primary clinical evidence of the CRS-associated phenotype was provided in a phase 1 study of 59 patients treated with CD22-directed CAR-T cells [53]. Among the 52 patients who developed CRS, about 40% met the study-defined criteria for CAR-associated HLH, characterized by a peak ferritin concentration of at least 100,000 µg/L together with at least two additional manifestations of severe organ toxicity, including coagulopathy, or hemophagocytosis [53]. The syndrome was characterized by hyperferritinemia, hypertriglyceridemia, hypofibrinogenemia, coagulopathy, hepatic transaminase elevation, hyperbilirubinemia, severe neutropenia, elevated lactate dehydrogenase, and occasional hemophagocytosis [53]. Because the study preceded the ASTCT definition and used different diagnostic criteria, the reported cases may not correspond directly to IEC-HS as currently defined. Nevertheless, the study provided primary evidence for an HLH-like inflammatory phenotype that develops in conjunction with CRS and is consistent with the current concept of CRS-MOD.

IEC-HS generally emerges within the first 3 weeks after CAR-T cell infusion, although the timing of onset may vary. The observational DESCAR-T registry study characterized 42 patients who developed IEC-HS after standard-of-care CD19-directed CAR-T cell therapy for B-cell lymphoma or B-cell acute lymphoblastic leukemia [55]. The median onset was 9 days after infusion, and the syndrome was characterized by marked hyperferritinemia, hypertriglyceridemia, elevated lactate dehydrogenase, and severe hypofibrinogenemia. Almost half of the patients had preceding grade 3-or-greater CRS, illustrating the close temporal relationship between the initial inflammatory response and subsequent IEC-HS, even though CRS and IEC-HS are clinically distinct [55].

A separate single-center study including 301 patients treated with CD19- or B-cell maturation antigen-directed commercial CAR-T cell products identified IEC-HS in almost 5% of patients [56]. IEC-HS developed at a median of 10 days after infusion and was accompanied by hyperferritinemia, neutropenia, thrombocytopenia, hepatic dysfunction, and pronounced peripheral CAR-T cell expansion. All affected patients had preceding CRS, and six also developed ICANS [56]. The study showed that IEC-HS can occur across different target antigens and various hematologic malignancies, including lymphoma, B-cell acute lymphoblastic leukemia, and multiple myeloma; however, the small number of affected patients limits meaningful comparisons by CAR-T cell product or disease type.

Severe IEC-HS may progress to worsening multilineage cytopenias, coagulopathy with hypofibrinogenemia, hepatic dysfunction, renal or pulmonary injury, hemodynamic instability, and multiorgan failure [4,54,55,56]. The key observational studies have shown all-cause mortality rates of about 80%, with many deaths being attributed directly to active IEC-HS [55,56]. However, these findings may be difficult to attribute specifically to IEC-HS, as patients also often had advanced hematologic malignancies, high disease burden, baseline cytopenias, preceding inflammatory toxicities, and frequent infections. Nevertheless, these reports show that IEC-HS is a distinct clinically consequential syndrome and not merely a transient biochemical extension of CRS.

Identifying IEC-HS is challenging because its clinicopathologic features are similar to those of persistent or recurrent CRS, infection, disease progression, immune effector cell-associated hematotoxicity, medication toxicity, and other causes of cytopenias or organ dysfunction. Infection, in particular, may not only mimic many features of IEC-HS but also can occur alongside the syndrome. In one retrospective study, infections occurred in 11 of 14 patients with IEC-HS, including four mixed infections [56]. Clinical assessment, therefore, is multifaceted and should consider the entire temporal course together with trends in ferritin trajectory, blood counts, fibrinogen, triglycerides, hepatic enzymes, coagulation parameters, and organ function. Overall, the distinction between the two CRS-associated phenotypes depends on whether HLH-like inflammation progresses during refractory CRS, which is consistent with CRS-MOD, or develops independently as CRS subsides, which aligns with IEC-HS [4,54].

#### 2.3.3. Biomarkers of IEC-HS

Studies evaluating biomarkers associated with IEC-HS have examined markers of systemic hyperinflammation, lymphoid and myeloid activation, hepatic injury, coagulation dysfunction, and hematopoietic suppression. For identification of IEC-HS, the ASTCT framework requires an elevated ferritin concentration defined as greater than twice the upper limit of normal or the patient’s baseline at the time of immune effector cell infusion and/or a rapidly rising ferritin concentration [4]. New or progressive cytopenias, hypofibrinogenemia and other coagulation abnormalities, hepatic transaminase elevation, hypertriglyceridemia, elevated lactate dehydrogenase, and organ dysfunction are other indicators. However, these features are nonspecific and may occur with CRS, infection, underlying malignancy, hepatic injury, and immune effector cell-associated hematotoxicity. Therefore, biomarker interpretation also depends on the timing, trajectory, and concurrence of abnormalities rather than any single laboratory value or threshold [4,5].

Previous framework identified ferritin as the central laboratory marker of IEC-HS, although its diagnostic specificity is currently limited, as studies have used a variety of approaches and definitions with no single established ferritin threshold available. For example, one study used an extreme peak ferritin concentration of at least 100,000 µg/L to define CAR-T cell-associated HLH following CD22-directed therapy [53]. In the DESCAR-T study, patients meeting the ASTCT criteria had median ferritin levels of over 18,000 µg/L [55]. And in another retrospective study, the median ferritin concentration at IEC-HS onset was a little over 15,000 µg/L in the setting of neutropenia, thrombocytopenia, hepatic dysfunction, and marked peripheral CAR-T cell expansion [56]; however, all 14 affected patients had baseline hyperferritinemia and cytopenias, highlighting the difficulty of interpreting post-infusion ferritin concentrations in isolation. The ASTCT Toxicity Consensus Group updated the ferritin elevation threshold requiring five-times increase from baseline or upper limit of normal [5]. However, there is no need to wait for all manifestations to be present, as a high index of suspicion for IEC-HS is important to start therapy and mitigate toxicity. Collectively, current evidence supports serial assessment of ferritin alongside other associated laboratory parameters for IEC-HS, but no established universal ferritin threshold is capable of definitively distinguishing IEC-HS from other post-infusion complications.

Mechanistically linked cytokine and cellular markers have also been investigated as potential markers for differentiating HLH-like toxicity from severe CRS. In a clinical trial of patients receiving CD22-directed CAR-T cells, HLH-like inflammation was associated with higher peak circulating interferon-γ, IL-1β, and IL-18 concentrations and persistent or recurrent peripheral CAR-T cell expansion [51]. Similar findings were observed in a syngeneic mouse model, in which perforin-deficient CAR-T cells underwent delayed re-expansion and generated an HLH-like inflammatory phenotype [45]. Additional clinical evidence came from a study of 99 patients who received B-cell maturation antigen-directed CAR-T cells, 20 of whom developed study-defined CAR-T cell-associated HLH-like toxicity [52]. Compared with severe CRS, the HLH-like phase was associated with greater elevations in interferon-γ, granzyme B, IL-1 receptor antagonist, and IL-10. The investigators also developed an exploratory model for distinguishing HLH-like toxicity from severe CRS by combining measurement of serum lactate dehydrogenase on day 6 and fibrinogen on day 3 after CRS onset. Results showed that the model had a good, but not excellent, ability to distinguish HLH-like toxicity from severe CRS [52]; however, the model is exploratory only, as it was derived from a single clinical trial, used criteria that predated the ASTCT definition, and has not been externally validated.

Soluble markers of macrophage and T-cell activation after CAR-T cell therapy have also been investigated as potential biomarkers for differentiating IEC-HS and CRS. In one small study, several possible diagnostic markers were serially measured in two patients who developed HLH-like toxicity after CD19-directed CAR-T cell therapy and four comparator control patients with differing patterns of CRS and cytopenia, including one patient with neither [57]. Because the six patients all had unique clinical manifestations, including only two with IEC-HS, the findings are limited. But overall, the authors noted that soluble CD163, CTLA4, CD80, and IL3345 were substantially increased in patients with IEC-HS and in those with CRS-macrophage activation syndrome relative to patients who had CRS alone, highlighting these as possible markers for distinguishing these entities from CRS and non-inflammatory new-onset cytopenia [57]. The authors described IEC-HS as an “elusive entity” and noted that the evaluated markers were primarily indicators of inflammatory severity rather than reliable diagnostic markers, emphasizing the need for more research to establish their diagnostic utility.

Established biomarkers for evaluating conventional HLH may also provide insight into the underlying mechanisms and clinical course of IEC-HS, but their utility in IEC-HS remains uncertain. CXCL9, which is an indirect marker of interferon-γ-mediated inflammation, is one such conventional HLH biomarker. In a multicenter retrospective study of 171 patients evaluated for HLH, 126, or about 74%, met the HLH-2004 and/or H-Score criteria [25]. Decision-tree analysis identified a CXCL9 concentration greater than 16,100 pg/mL as the optimal laboratory predictor of inpatient mortality. The same threshold remained associated with 90-day mortality after adjustment, retaining prognostic value when patients were stratified by malignancy-associated and non-malignancy-associated HLH [25]. Although interferon-γ signaling is biologically relevant to IEC-HS, CXCL9 has not yet been validated as an IEC-HS-specific diagnostic or prognostic marker (Table 1).

Soluble CD25 and ferritin have similarly been combined to identify clinically consequential hyperinflammation associated with hematologic malignancy. In a cohort of 225 patients with hematologic malignancies, these biomarkers were used to develop an optimized HLH inflammatory index [58]. The study identified thresholds of soluble CD25 greater than 3900 U/mL and ferritin greater than 1000 ng/mL for identifying patients meeting the HLH-2004 criteria with 84% sensitivity and 81% specificity. Moreover, the index was associated with increased mortality across malignancy groups [58]. However, the study addressed malignancy-associated HLH rather than IEC-HS following CAR-T cell therapy. Recently, IEC-HS that occurred in 19 of 54 patients with B-cell malignancy who received CD22 CAR-T cell was reported [59]. While IEC-HS was tied to greater CAR-T cell expansion and a distinct cytokine signature, its severity was most strongly associated with elevated levels of three specific cytokines: IFNγ, IL10, and IL1RA. Therefore, while the index supports the potential value of combining a marker of T-cell activation with ferritin, it cannot yet be directly applied to IEC-HS without validation in patients who have overlapping CRS, baseline cytopenias, lymphodepletion-related hematotoxicity, and post-infusion inflammation.

In summary, no individual biomarkers or validated panel of markers currently has the power to reliably distinguish IEC-HS from severe or recurrent CRS, infection, disease progression, hepatic injury, or immune effector cell-associated hematotoxicity. Serial ferritin, blood counts, fibrinogen, triglycerides, lactate dehydrogenase, hepatic enzymes, and coagulation parameters remain the most clinically accessible measures of evolving disease. Cytokine profiles, soluble immune-activation markers, CXCL9, soluble CD25, and peripheral CAR-T cell expansion may improve biologic characterization and risk stratification, but their diagnostic thresholds, optimal sampling intervals, and performance across CAR-T cell constructs and malignancies require prospective multicenter validation. Cytokine concentrations are not currently included in the ASTCT diagnostic framework, and identification of IEC-HS should remain based on careful analysis of the overall pattern of clinical and laboratory findings rather than on any single parameter [4].

## 3. Conclusions and Future Directions

The therapeutic advantages of adoptive cellular immunotherapy cannot be separated from the potential risk of severe hyperinflammatory toxicities. Unraveling the molecular mechanisms underlying CRS, ICANS, and IEC-HS will help enable the development of strategies to mitigate these toxicities while preserving the therapeutic effectiveness of CAR-T cell therapies. A deeper understanding of these toxicities may also inform the development of predictive models for identifying patients most at risk for these dangerous complications, as well as engineered cellular approaches designed to control inflammatory responses. Mechanistic insight into CAR-T cell therapy-associated toxicities and reliable methods for identifying and differentiating them will be critical for moving beyond treatment of established toxicity toward individualized strategies for prevention and early intervention.

## Figures and Tables

**Table 1:** Some of the biomarker strategies for identifying CAR-T cell therapy-associated toxicities.

Biomarker/Risk Score	Formula and Biologic Components	Clinical Utility and Predictive Value in Cellular Therapy	Reference
EASIX	(LDH × Creatinine)/Platelets	Baseline score correlates with underlying endothelial injury but lacks specificity for acute inflammatory bursts	[12]
m-EASIX	Log2 ((CRP × Ferritin)/Platelets))	Calculates risk before infusion and 1 and 3 days after infusion	[12,13]
Inflammatory Cytokines	Peak levels of IFN-*γ*, IL-6, IL-10, IL-15, TNF-*α*	Correlates with early toxicity. Slow laboratory turnaround times limit real-time clinical utility	[22]
Endothelial Markers	Angiopoietin-2, VCAM-1, von Willebrand factor	Reflects systemic capillary leak, widespread coagulopathy, and BBB disruption. Elevated Ang-2-to-Ang-1 ratio is strongly implicated in the pathogenesis of severe ICANS	[12]
Glial/Axonal Markers	GFAP, NfL, S100 proteins	Elevated CSF and serum levels indicate direct CNS involvement and profound neuroaxonal injury at time of ICANs	[23,24]
CXCL9	Continuous increase in CXCL9	Continuous increases in CXCL9 associated with greater mortality	[25]

Ang: angiopoietin; BBB: blood–brain barrier; CRP: C-reactive protein; GFAP: glial fibrillary acidic protein; ICANS: immune effector cell-associated neurotoxicity syndrome; IFN: interferon; IL: interleukin; LDH: lactate dehydrogenase; NfL: neurofilament light chain; TNF: tumor necrosis factor; VCAM-1: vascular cell adhesion molecule-1.

## Data Availability

No new data were created or analyzed in this study. Data sharing is not applicable to this article.

## Abbreviations

The following abbreviations are used in this manuscript:

ASTCT	American Society for Transplantation and Cellular Therapy
CAR-T	Chimeric antigen receptor T cell
CNS	Central nervous system
CRP	C-reactive protein
CRS	Cytokine release syndrome
CRS-MOD	Cytokine release syndrome–multiorgan dysfunction
CTCAE	Common Terminology Criteria for Adverse Events
EEG	Electroencephalography
GM-CSF	Granulocyte-macrophage colony-stimulating factor
HLH	Hemophagocytic lympho-histiocytosis
ICANS	Immune effector cell-associated neurotoxicity syndrome
IEC-HS	Immune effector cell-associated hemophagocytic lympho-histiocytosis-like syndrome
IFN	Interferon
IL	Interleukin
IV	Intravenous
LDH	Lactate dehydrogenase
LVEF	Left ventricular ejection fraction
MRI	Magnetic resonance imaging
NCCN	National Comprehensive Cancer Network
TNF	Tumor necrosis factor
